# Association of Alzheimer’s disease polygenic risk score with concussion severity and recovery metrics

**DOI:** 10.1002/alz.090799

**Published:** 2025-01-09

**Authors:** Kaitlyn M Dybing, Thomas W McAllister, Yu‐Chien Wu, Brenna C. McDonald, Michael A. McCrea, Steven P. Broglio, Paul F. Pasquina, M. Alison Brooks, Jason P. Mihalik, Kevin M Guskiewicz, Christopher C. Giza, Joshua Goldman, Stefan Duma, Steve Rowson, Steven Svoboda, Kenneth L. Cameron, Megan N. Houston, Darren E. Campbell, Gerald McGinty, Jonathan Jackson, Shannon L. Risacher, Andrew J. Saykin, Kelly N. Nudelman

**Affiliations:** ^1^ Indiana University School of Medicine, Indianapolis, IN USA; ^2^ Indiana University/School of Medicine, Indianapolis, IN USA; ^3^ Stark Neurosciences Research Institute, Indiana University School of Medicine, Indianapolis, IN USA; ^4^ Indiana Alzheimer’s Disease Research Center, Indiana University School of Medicine, Indianapolis, IN USA; ^5^ Indiana University Network Science Institute, Bloomington, IN USA; ^6^ Indiana Alzheimer’s Disease Research Center, Indianapolis, IN USA; ^7^ Medical College of Wisconsin, Milwaukee, WI USA; ^8^ University of Michigan, Ann Arbor, MI USA; ^9^ Uniformed Services University of the Health Sciences, Bethesda, MD USA; ^10^ University of Wisconsin School of Medicine and Public Health, Madison, WI USA; ^11^ The University of North Carolina, Chapel Hill, NC USA; ^12^ University of North Carolina at Chapel Hill, Chapel Hill, NC USA; ^13^ David Geffen School of Medicine at UCLA, Los Angeles, CA USA; ^14^ University of California, Los Angeles, Los Angeles, CA USA; ^15^ Virginia Tech, Blacksburg, VA USA; ^16^ United States Military Academy, West Point, NY USA; ^17^ United States Air Force Academy, Air Force Academy, CO USA; ^18^ Center for Neuroimaging, Department of Radiology and Imaging Sciences, Indiana University School of Medicine, Indianapolis, IN USA; ^19^ Indiana Alzheimer's Disease Research Center, Indiana University School of Medicine, Indianapolis, IN USA; ^20^ Center for Computational Biology and Bioinformatics, Indiana University School of Medicine, Indianapolis, IN USA; ^21^ Indiana University, Indianapolis, IN USA; ^22^ National Centralized Repository for Alzheimer’s Disease and Related Dementias (NCRAD), Indianapolis, IN USA; ^23^ Department of Medical and Molecular Genetics, Indiana University School of Medicine, Indianapolis, IN USA

## Abstract

**Background:**

Shared genetic risk between Alzheimer’s disease (AD) and concussion may help explain the association between concussion and elevated risk for dementia. However, there has been little investigation into whether AD risk genes also associate with concussion severity/recovery, and the limited findings are mixed. We used AD polygenic risk scores (PRS) and *APOE* genotypes to investigate associations between AD genetic risk and concussion severity/recovery in the NCAA‐DoD Grand Alliance CARE Consortium (CARE) dataset.

**Method:**

There were 1,917 injuries in the dataset upon project initiation. After removing repeated injuries, related participants, and those without genetic/outcome data, we had 931 participants. Outcomes were number of days to return to play (RTP) as a recovery measure, and four severity measures (scores on SAC and BESS, SCAT symptom severity and total number of symptoms). We calculated PRS using a published score (de Rojas et al., 2021) and performed a linear regression (MLR) of RTP by PRS in normal (<24 days) and long (>24 days) RTP subgroups. We then compared severity measures by PRS using MLR. Next, we used t‐tests to examine outcomes by *APOE* genotype in military and civilian subgroups. We also performed chi‐squared tests of RTP category (normal vs. long) by *APOE* genotype. Finally, we analyzed outcomes by PRS in European or African genetic ancestry subgroups using MLR.

**Result:**

Higher PRS was associated with longer injury to RTP interval in the normal RTP (<24 days) subgroup (estimate = 0.0412, SE = 0.182, *p* = 0.0237). 1 SD increase in PRS resulted in a 0.412 day (9.89 hours) increase to the interval. This may be clinically meaningful in the collegiate athlete environment. We did not identify any other significant differences.

**Conclusion:**

Our preliminary results provide limited evidence for an impact of AD PRS on concussion recovery, though the pattern was inconsistent and its clinical significance is uncertain. Future studies should attempt to replicate these findings in larger samples with longer follow‐up using PRS calculated from multiple/diverse populations, which will be especially relevant for diverse datasets like CARE.

